# Association between baseline dissociation levels and stress-induced state dissociation in patients with posttraumatic-stress disorder, borderline personality disorder, and major depressive disorder

**DOI:** 10.1186/s40479-023-00215-2

**Published:** 2023-03-30

**Authors:** Livia Graumann, Johannes Bodo Heekerens, Moritz Duesenberg, Sophie Metz, Carsten Spitzer, Christian Otte, Stefan Roepke, Katja Wingenfeld

**Affiliations:** 1 Department of Psychiatry and Neuroscience, Charité – Universitaetsmedizin Berlin, corporate member of Freie Universitaet Berlin, Humboldt- Universitaet Zu Berlin, and Berlin Institute of Health, Campus Benjamin Franklin, Berlin, Germany; 2 Department of Medical Psychology, Charité – Universitaetsmedizin Berlin, corporate member of Freie Universitaet Berlin, Humboldt- Universitaet Zu Berlin, and Berlin Institute of Health, Berlin, Germany; 3 Department of Psychosomatic Medicine, Rostock University Medical Center, Rostock, Germany

**Keywords:** Borderline personality disorder, Posttraumatic-stress disorder, Major depressive disorder, Dissociation, Psychosocial stress, Trier Social Stress Test, Structural equation modeling

## Abstract

**Introduction:**

Dissociative symptoms are highly prevalent in patients with trauma-related disorders such as borderline personality disorder (BPD) and posttraumatic-stress disorder (PTSD), and also occur in patients with depressive disorders. Acute dissociative states are theorized to be stress-related, and some individuals experience recurring patterns of dissociation. The relationship between the intensity of dissociative episodes (trait-like dissociation) and acute dissociative states, however, is incompletely understood. In the present study, we investigated how levels of baseline (trait-like) dissociation relate to changes in dissociative states during a laboratory stress induction.

**Methods:**

Our female sample comprised 65 patients with BPD and/or PTSD, 84 patients with major depressive disorder (MDD) and 44 non-clinical controls (NCC). Baseline dissociation was assessed at the start of the study using the Dissociation Tension Scale past week version (DSS-7). All participants underwent the Trier Social Stress Test (TSST) and a placebo version (P-TSST). Before and after the TSST or P-TSST, state dissociation was assessed using the Dissociation Tension Scale acute (DSS-4). We used structural equation models to estimate changes in state dissociation items (somatoform dissociation, derealization, depersonalization, analgesia), and to test whether these changes relate to levels of baseline dissociation.

**Results:**

We found significant increases in all state dissociation items in response to the TSST in patients with BPD and/or PTSD and patients with MDD, but not in NCCs. Increases in somatoform dissociation and derealization during the TSST were significantly related to higher levels of baseline dissociation in patients with BPD and/or PTSD, but not in patients with MDD or NCCs. Results indicate no significant changes in state dissociation during the P-TSST.

**Conclusion:**

Our results replicate earlier findings that patients with BPD and/or PTSD report higher levels of stress-related state dissociation than NCC and extend them to patients with MDD. In addition, our findings indicate that baseline levels of dissociation relate to stress-induced changes in state dissociation among patients with BPD and PTSD, but not patients with MDD. In clinical applications, measures of baseline dissociation could be used to facilitate the prediction and treatment of stress-related dissociative states in patients with BPD and/or PTSD.

**Supplementary Information:**

The online version contains supplementary material available at 10.1186/s40479-023-00215-2.

## Background

Dissociation is a multifaceted psychological phenomenon, which has been observed in various mental disorders [1] and is discussed as a transdiagnostic marker of psychopathology [2]. It is defined as a “disruption of and/or discontinuity in the normal integration of consciousness, memory, identity, emotion, perception, body representation, motor control, and behavior” [3] (p. 330). Dissociative disorders such as depersonalization/derealization disorder, dissociative identity disorder, and dissociative amnesia are primarily characterized by dissociation, and dissociative symptoms also serve as a criterion for borderline personality disorder (BPD) and the dissociative subtype of posttraumatic-stress disorder (PTSD) [3]. Dissociation is also reported by individuals with depressive disorders [1] and individuals from the general population with depressive symptoms [4]. Dissociative symptoms frequently observed in clinical practice include depersonalization (e.g., feeling detached from the own body), derealization (e.g., experiences of unreality of surroundings), somatoform dissociation (e.g., difficulties hearing), and analgesia to pain [5, 6].

While acute dissociative symptoms (state dissociation) are typically short-lived and frequently occur in response to stress, some individuals experience recurrent patterns of dissociative symptoms [3]. Such patterns have been conceptualized as a relatively stable disposition (trait dissociation) or episodes of limited duration (trait-like dissociation). Related questionnaires ask patients to indicate the amount of time they generally have dissociative experiences in their everyday life (trait dissociation) (e.g., Dissociative Experience Scale) [7] or the amount of time they had dissociative experiences during a fixed period, usually one week (trait-like dissociation) (e.g., Dissociation Tension Scale past week version) [8]. Studies suggests that patients with BPD and/or PTSD experience higher levels of trait and trait-like dissociation than non-clinical controls and other clinical groups including patients with depressive disorders [1]. In addition, dissociation has been linked to a variety of adverse mental health outcomes, such as more psychopathological symptoms in patients with PTSD [9], as well as non-suicidal self-injury and suicide attempts in patients with BPD [10, 11]. This points to the importance of dissociative phenomena in clinical research and practice. However, the distal and focal factors influencing the development and maintenance of dissociative symptoms are still debated [12, 13]. Specifically, the relationship between baseline levels of dissociation and acute stress-induced state dissociation is incompletely understood. In this study, we investigate the relationship between dissociation at baseline and dissociative states following a stress induction in a sample of patients with BPD and/or PTSD, and compare those to clinical control sample of patients with major depressive disorder (MDD) and non-clinical control (NCC) participants.

### Links between trauma, stress, and dissociation

According to trauma models, dissociative states are an initially adaptive form of coping with overwhelming and stressful experiences, especially when there are low chances to escape [14–16]. Such peri-traumatic dissociative states can include alterations in perception of time, place, and self (e.g., in the form of depersonalization or derealization), which may function to subjectively detach from the traumatic event and associated feelings of distress. Meta-analytic findings show that individuals who report higher levels of retrospective childhood abuse, especially sexual and physical abuse, report more dissociation in adulthood than individuals with lower levels of retrospective childhood abuse [17]. Experiences of childhood abuse are also related to general psychopathology including BPD and PTSD [18, 19].

Recurring dissociative symptoms can be understood as a function of classical and operant conditioning [20, 21]. After an initial association with trauma-related stimuli, dissociative reactions might generalize to other (unspecific) stressors resulting in a heightened predisposition for dissociation. Individuals high in trait or trait-like dissociation (e.g., patients with BPD and/or PTSD) may then frequently react to stress with dissociative states (conditioned response). In line with this, cross-sectional associations between measures of trait and state dissociation have been reported in patients with PTSD [22], patients with BPD [23] and a non-clinical sample of police officers [24]. This association does not seem to be limited to trauma-related stressors only, but extends to other types of stressors as well. Ebner-Priemer et al. [23] report higher state dissociation in response to an emotional learning task in patients with BPD with higher trait dissociation. Zoellner, Sacks, and Foa [22] found increased state dissociation among patients with PTSD with higher trait dissociation after confrontation with a dissociation induction with own memories of detachment from non-traumatic emotional situations. In a sample of police recruits [24], general life stress accounted for a significant amount of variance in dissociation and PTSD symptoms beyond that accounted for by the number of traumatic events only.

In addition, several studies support the link between subjective distress and state dissociation among patients with BPD and PTSD. Specifically, studies found positive cross-sectional associations between subjective distress and state dissociation in patients with BPD using retrospective reports [25], as well as using multiple momentary assessments in everyday life in patients with BPD and PTSD, but not in non-clinical controls [26]. Furthermore, patients with BPD reported higher levels of dissociative symptoms relative to subjective stress ratings than clinical and non-clinical controls [26]. Using multiple momentary assessments in everyday life, another study found that increases in unpleasant arousal preceded dissociative states in patients with BPD, but not in non-clinical controls or patients with depressive disorders [21].

To our knowledge, only few studies have investigated dissociative reactions in response to experimental stressors and current findings are mixed. A frequently used psychosocial stressor is the Trier Social Stress Test (TSST) [27], which has been shown to result in heightened psychological arousal and distress among traumatized patients [28, 29]. In a sample of patients with BPD, Scott et al. [30] found no differences in dissociative reactions to psychosocial stress induced with the TSST compared to non-clinical controls, while Zaba et al. [31] found higher state dissociation after TSST among patients with PTSD compared to non-clinical controls. In another TSST study, patients with BPD who scored higher in trait dissociation showed a more pronounced stress response than those lower in trait dissociation, as indicated by heightened plasma cortisol levels [32]. Similar results were reported in patients with PTSD after recounting traumatic experiences [33].

In sum, current evidence suggests that acute stress relates to higher levels of state dissociation in patients with BPD and/or PTSD but not non-clinical individuals and other clinical groups, such as patients with depressive disorders. In addition, state dissociation has been associated with trait dissociation, and levels of trait dissociation relate to retrospective self-reports of childhood abuse. However, comparisons of acute dissociation after a stress induction to acute dissociation after a non-stressful control condition are currently missing, as well as studies on the direct link between experimentally induced stress-related state dissociation and trait dissociation.

### Study aim and post-hoc hypotheses

The aim of this study was to investigate the association between baseline dissociation and stress-induced state dissociation in two patient samples (BPD / PTSD and MDD) and in non-clinical controls. We expected that higher baseline values in trait-like dissociation predict stronger increases in dissociative states among patients with BPD and/or PTSD, but not patients with MDD and NCCs.

## Method

### Data transparency and code availability

The data reported in this manuscript were collected in two separate and previously published studies. The BPD/PTSD and NCC samples are described in Duesenberg et al. [34], and Metz et al. [35]. The MDD sample is described in Wingenfeld et al. [36]. The current manuscript focuses on the relation between baseline dissociation and dissociative states following a stress induction. Only those methods and results relevant for our research questions will be described in detail in this manuscript. We are unable to share any data publicly because participants were not explicitly asked to agree to make their anonymized data available online. Thus, sharing participants’ data would violate confidentiality. Anonymized data sets will be made available to researchers upon request. Statistical code and (additional) results for this manuscript are available at https://osf.io/qnmkf.

### Procedure

Participants in this study were recruited at Charité – Universitaetsmedizin Berlin, and by local and online advertisements (BPD/PTSD and NCC samples; for details, see [34, 35]), as well as at Charité – Universitaetsmedizin Berlin and Fachklinikum Tiefenbrunn (MDD sample; for details, see [36]). All participants gave written informed consent prior to participation. We received approval by the Ethics committee of the Charité – Universitaetsmedizin Berlin.

**Fig. 1:**
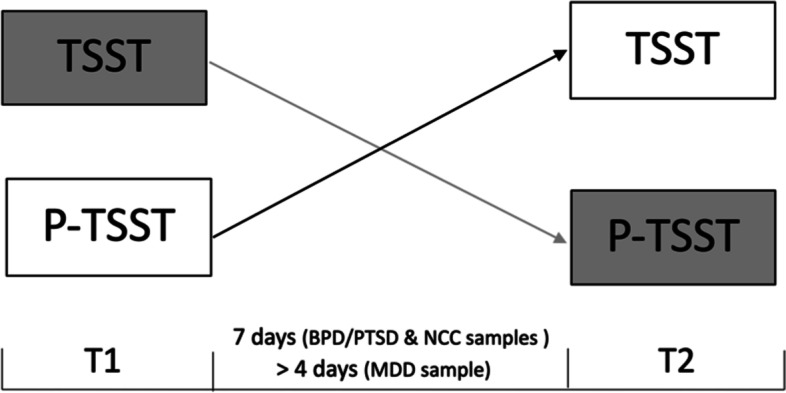
*Crossover Study Design* TSST = Trier Social Stress Test; P-TSST = Placebo Trier Stress Test; T1 = testing session one; T2 = testing session two; BPD = borderline personality disorder; PTSD = posttraumatic-stress disorder; MDD = major depressive disorder; NCC = non-clinical controls

A shown in Fig. 1, all participants underwent two testing sessions held in laboratory rooms. Participants were randomly assigned to either first undergo a psychosocial stressor (TSST) and then, approximately one week later (BPD/PTSD and NCC samples) or more than four days later (MDD sample), a non-stressful control condition (P-TSST), or first a non-stressful control condition and then a psychosocial stressor (cross-over design).

### Experimental stress induction

The Trier Social Stress Test (TSST) was used to induce stress [27]. The study investigator instructed participants to prepare for a job interview (5 min) and to present themselves in front of a committee of two members, a camera and microphone for voice recording (5 min). Participants were told that these committee members were trained behavioral analysts and that the camera and voice recorder would record their presentation for later analysis. Afterwards, one of the committee members instructed participants to carry out a difficult arithmetic task (5 min.). As a control condition, we used a “Placebo” version of the TSST (P-TSST) [37]. After a preparation phase, participants were instructed to talk aloud about a topic of choice in an empty room. Subsequently, participants performed an easy arithmetic task (5 min).

### Participants

In- and exclusion criteria for all participants were assessed by trained clinicians using the German version of the Structured Clinical Interview for DSM-IV axis I and II (SCID) [38, 39]. Non-clinical control participants had to be free of any current or past psychiatric disorders. Exclusion criteria for the patient groups were schizophrenia, schizoaffective disorder, bipolar disorder, anorexia, alcohol or drug abuse and dependence in the last six months. We did not include patients with BPD and/or PTSD who were diagnosed with a comorbid current major depressive disorder. In the MDD group, there were no patients with a comorbid PTSD, but two patients with a comorbid BPD diagnosis. We decided not to exclude these patients because at the time of enrollment the MDD was the primary diagnosis and the target of treatments.

For the present analysis, we included all participants who provided answers on the Dissociation Tension Scale (DSS-7) [8] as a measure of baseline or trait-like dissociation, as well as the Dissociation Tension Scale acute (DSS-4) [5] as a measure of state dissociation. Our female sample comprised 65 BPD / PTSD patients (25 BPD, 20 BPD & PTSD, 20 PTSD), 84 patients with MDD and 44 NCCs. For a detailed record of comorbid diagnoses and psychotropic substances for both patient groups, see supplement material S1. Types and frequencies of reported traumatic experiences among the BPD/PTSD group are listed in supplement material S2. Participant flow for each sample is depicted and reported in supplement material S3. As shown in Table 1., analysis of variance and Pearson’s chi-squared results show no significant differences in age or years of education across diagnostic groups (BPD / PTSD, MDD, and NCC).

**Table 1.** Participant Characteristics in Patients with BPD and/or PTSD (*n* = 65), Patients with MDD (*n* = 84), and Non-Clinical Controls (*n* = 41)

**Variable ** *(M/SD)*	**BPD/PTSD Patients**	**MDD Patients**	**Non-Clinical Controls**	**Test statistic**	**Effect size**
Age	31.88 (8.80)	34.99 (11.22)	32.20 (9.19)	*F*(2, 190) = 2.12, *p* = .123	*η*^*2*^ = 0.02, 90% CI [0, 0.06]
Years of school education	11.43 (1.78)	11.41(1.47)	11.70 (1.42)	*F*(2, 186) = 0.55, *p* = .577	*η*^*2*^ = 0.01, 90% CI [0, 0.03]
Baseline dissociation (DSS-7)	28.43 (17.80)	13.78 (13.15)	2.94 (4.40)	*F*(2, 186) = 45.78, *p* < .001	*η*^*2*^ = 0.33, 90% CI [0.24, 0.41]
Childhood maltreatment (CTQ)	68.21 (19.10)	50.07 (16.40)	32.85 (7.81)	*F*(2, 187) = 62.66, *p* < .001	*η*^*2*^ = 0.40, 90% CI [0.31, 0.47]
Depressive symptom severity (BDI-II)	27.30 (10.23)	22.06 (10.83)	2.61 (2.79)	*F*(2, 175) = 90.42, *p* < .001	*η*^*2*^ = 0.51, 90% CI [0.42, 0.57]

*Note.* We report sum scores for all questionnaires. *BPD* borderline personality disorder, *PTSD *posttraumatic-stress disorder, *MDD *major depressive disorder, *M *mean, *SD *standard deviation, *η*^*2*^ eta squared (effect size), *DSS-7* Dissociation Tension Scale (past week), *CTQ *Childhood Trauma Questionnaire, *BDI-II *Beck Depression Inventory, Post-hoc t-test results: baseline dissociation: BPD/PTSD > MDD: *t*(146) = 5.76, *p* < .001, BPD/ PTSD > NCC: *t*(103) = 8.98, *p* < .001, MDD > NCC: *t*(123) = -5.13, *p* < .001; childhood maltreatment: BPD/PTSD > MDD: *t*(144) = 6.02, *p* < .001, BPD/PTSD > NCC: *t*(106) = 11.15, *p* < .001, MDD > NCC: *t*(124) = -6.58*, p* < .001; depressive symptom severity: BPD/PTSD > MDD: *t*(135) = 2.88, *p* = .005, BPD/ PTSD > NCC: *t*(100) = 15.06, *p* < .001, MDD > NCC,* t*(115) = -11.28, *p* < .001

### Measures

#### Baseline measures

Participants filled out the following questionnaires before the intervention at the first testing session. Means, standard deviations and statistics are listed in Table 1. A detailed description of all measures is included in Duesenberg et al. [34] and Wingenfeld et al. [36].

##### Baseline dissociation

To assess severity of dissociative symptoms in the week prior to the experiment, we used the German Dissociation Tension Scale (DSS-7) [8]. Participants rated how often they experienced each of 21 sensations (e.g., “I couldn’t feel my body or parts of it”) within the last seven days on a scale ranging from 0 (*never*) to 100 (*all of the time*). Mc Donald’s (1999) hierarchical omega was 0.96, 95% CI [0.94, 0.97] (confidence intervals were calculated using bias-corrected and accelerated bootstraps as implemented in the R package MBESS) [40].

##### Childhood maltreatment

We used the German version of the Childhood Trauma Questionnaire (CTQ) [41, 42] to retrospectively assesses childhood maltreatment using 28 items (e.g., “When I was growing up, I didn’t have enough to eat”). The scale ranges from 1 (*not at all*) to 5 (*very often*). Mc Donald’s hierarchical omega was 0.94, 95% CI [0.92, 0.96].

##### Depressive symptom severity

Depressive symptoms within the last two weeks were assessed using the German version of the revised Beck Depression Inventory (BDI-II) [43, 44]. The BDI-II includes 21 items relating to symptoms of depression. Participants indicate the degree of severity for each symptom on a scale ranging from 0 to 3. Hierarchical omega was 0.94, 95% CI [0.93, 0.96].

#### Repeated measures

Participants filled out the following questionnaires immediately before and after the intervention (TSST or P-TSST) at each testing session.

##### State dissociation

We administered the German Dissociation Tension Scale acute (DSS-4) [5]. The scale assesses four clinically significant facets of current dissociative experiences using one item for each facet from 0 (*not at all*) to 9 (*very much*) scale. The scale assesses depersonalization, (“I have the impression that my body does not belong to me”), somatoform dissociation (“I have problems hearing, e.g. I hear sounds from nearby as if they come from far away”), derealization (“I have the impression other people or things around me are unreal”), and analgesia (“I have the impression that my body or parts of it are insensitive to pain”). For the original German wording, see supplement material S4.

##### Affect

Also immediately before and after the (P-)TSST, we used the short version A of the German Multidimensional Mood State Questionnaire (MDBF) [45, 46] to assess affective states. The scale includes four items each referring to positive–negative mood (e.g., “happy”), awake-tired mood (e.g., “rested”), and calm-agitated mood (e.g., “restless”). Participants were asked how they feel “at the moment”. The scale is anchored at 1 (*not at all*) and 5 (*very*).

### Statistical analysis

We analyzed our data using structural equation models that allow us to test underlying assumptions of longitudinal models (e.g., measurement equivalence) [47]. In this study, we were interested in changes in dissociative states across two measurement occasions (immediately before vs. immediately after the TSST or P-TSST). On each measurement occasion, dissociative states were assessed using four observed variables (indicators or items) that were expected to load on a common factor [6]. Because meaningful across-time comparisons (e.g., of latent means) require strong scale invariance (equal item loadings and intercepts) across time, we tested this prerequisite in a first step of our analysis [47, 48]. For this purpose, we defined a model assuming that a single factor is present at each measurement occasion and that all four items load onto the same factors across time (configural scale invariance). Results show that this model, which is less restrictive than the strong scale invariant model because item loadings and intercepts are freely estimated across time, is an inappropriate fit across patients with BPD and/or PTSD in the TSST condition, ${\chi }^{2}$(19, *N* = 64) = 112.10, *p* = 0.000, CFI = 0.75, RMSEA = 0.28, 95% CI [0.23, 0.33], SRMR = 0.10, and the P-TSST condition, ${\chi }^{2}$(25, *N* = 61) = 94.81, *p* = 0.000, CFI = 0.75, RMSEA = 0.25, 95% CI [0.20, 0.31], SRMR = 0.10 (as indicated by CFI < 0.97, RMSEA > 0.05, and SRMR $\ge$ 0.08; [49, 50]. Thus, the prerequisite of strong scale invariance across time is likely violated in our data, suggesting that latent means in state dissociation cannot be meaningfully compared across time points. For this reason, we decided to investigate changes in the four items (depersonalization, somatoform dissociation, derealization, and analgesia) rather than change in a single dissociative states factor. This approach aligns well with the theoretical definition of dissociation as a multifaceted construct [3]. Specifically, we defined item-specific linear growth models as multilevel models [51, 52] with two measurement occasions that specify time-invariant intercepts and linear slopes at the between-person level, as well as residual variables at the within-person level. Each dissociative states item is linearly regressed on time (dummy coded: 1 = immediately before the lab session, 2 = immediately after the lab session) and random intercepts and random slopes are estimated for each item. We computed a total of six models under the TSST and P-TSST conditions in patients with PTSD and/or BPD, patients with MDD, and NNCs. The models regressed the random intercepts and linear slopes on baseline dissociation to test whether changes in items depend on observed baseline dissociation scores. Table 2 shows that the models estimate average intercepts and slopes in state dissociation items (fixed effects), as well as person-specific deviations from these averages (random-effects variances). The intercepts capture state dissociation scores before the stress induction (or placebo), whereas the slopes capture change during the stress induction (or placebo). The models also estimate the degree to which differences in baseline dissociation predict differences in intercepts and slopes. The estimation of our models is based on Bayesian methods because other than maximum likelihood approaches, this allowed us to judge the appropriateness of our models in M*plus* [53]. Specifically, model convergence was assumed if the potential scale reduction factor fell below the M*plus* default cut-off of 1.10 for all parameters, and after careful inspection of trace plots. All models converged well. Bayesian analyses were conducted using the M*plus* default priors.

**Table 2:** Results of Structural Equation Models of Baseline Levels and Changes in Dissociative States Items in Stress and Non-Stress Conditions in Patient and Non-Clinical Samples Predicted by Baseline Dissociation

	Borderline Personality and/or Posttraumatic Stress Disorder				Major Depressive Disorder				Non-Clinical Controls			
	Stress Condition (TSST)		Non-Stress Condition (P-TSST)		Stress Condition (TSST)		Non-Stress Condition (P-TSST)		Stress Condition (TSST)		Non-Stress Condition (P-TSST)	
	*n* = 64		*n* = 62		*n* = 84		*n* = 84		*n* = 43		*n* = 44	
	Estimate	95% CI	Estimate	95% CI	Estimate	95% CI	Estimate	95% CI	Estimate	95% CI	Estimate	95% CI
Fixed effects (average effects across participants)												
*Intercepts (before stress induction)*												
$\gamma _{\alpha _{depersonalization}}$	1.54	[1.06, 2.08]	1.67	[1.13, 2.22]	0.93	[0.51, 1.34]	1.04	[0.72, 1.35]	0.12	[-0.02, 0.25]	0.11	[0.01, 0.22]
$\gamma _{\alpha _{somatoform}}$	1.26	[1.06, 2.08]	1.67	[1.08, 2.24]	0.49	[0.25, 0.71]	0.69	[0.40, 0.97]	0.23	[0.02, 0.45]	0.09	[-0.02, 0.20]
$\gamma _{\alpha _{derealization}}$	1.51	[1.02, 1.99]	1.43	[0.90, 1.97]	0.66	[0.33, 0.99]	0.96	[0.65, 1.27]	0.07	[-0.02, 0.17]	0.05	[-0.02, 0.11]
$\gamma _{\alpha _{analgesia}}$	1.98	[1.42, 2.56]	2.19	[1.56, 2.86]	0.75	[0.40, 1.10]	0.85	[0.53, 1.16]	0.12	[-0.01, 0.24]	0.20	[-0.07, 0.47]
*Slopes (change during stress induction)*												
$\gamma _{\beta _{depersonalization}}$	1.46	[0.87, 2.07]	0.12	[-0.50, 0.67]	0.69	[0.19, 1.18]	-0.09	[-0.40, 0.21]	0.09	[-0.12, 0.31]	-0.11	[-0.23, 0.01]
$\gamma _{\beta _{somatoform}}$	0.92	[0.43, 1.41]	-0.08	[-0.60, 0.47]	0.86	[0.43, 1.27]	-0.03	[-0.32, 0.26]	0.03	[-0.17, 0.22]	-0.05	[-0.17, 0.08]
$\gamma _{\beta _{derealization}}$	1.04	[0.59, 1.48]	0.17	[-0.37, 0.67]	0.94	[0.47, 1.42]	-0.15	[-0.43, 0.15]	0.14	[-0.01, 0.28]	-0.02	[-0.10, 0.06]
$\gamma _{\beta _{analgesia}}$	0.87	[0.45, 1.30]	-0.26	[-0.90, 0.36]	0.61	[0.22, 1.01]	-0.06	[-0.34, 0.24]	0.02	[-0.13, 0.18]	-0.09	[-0.24, 0.05]
Random-effects variances (differences across participants)												
*Intercepts (before stress induction)*												
$\varphi _{\alpha _{depersonalization}}$	1.54	[0.56, 3.11]	2.07	[1.00, 3.78]	1.80	[0.90, 3.07]	1.18	[0.70, 1.90]	0.15	[0.06, 0.28]	0.04	[0.01, 0.10]
$\varphi _{\alpha _{somatoform}}$	2.21	[1.19, 3.81]	3.38	[1.92, 5.68]	0.47	[0.18, 0.89]	0.76	[0.39, 1.28]	0.29	[0.14, 0.57]	0.05	[0.02, 0.12]
$\varphi _{\alpha _{derealization}}$	2.54	[1.53, 4.24]	2.57	[1.43, 4.45]	0.73	[0.24, 1.48]	1.15	[0.69, 1.80]	0.06	[0.02, 0.12]	0.01	[0.00, 0.03]
$\varphi _{\alpha _{analgesia}}$	4.52	[2.94, 7.18]	3.90	[2.14, 6.69]	1.42	[0.76, 2.38]	1.28	[0.77, 1.98]	0.08	[0.03, 0.19]	0.72	[0.43, 1.28]
*Slopes (change during stress induction)*												
$\varphi _{\beta _{depersonalization}}$	0.85	[0.05, 2.64]	0.29	[0.01, 1.30]	1.58	[0.45, 3.04]	0.12	[0.01, 0.50]	0.38	[0.20, 0.67]	0.01	[0.00, 0.04]
$\varphi _{\beta _{somatoform}}$	0.47	[0.03, 1.67]	0.33	[0.02, 1.55]	2.27	[1.45, 3.42]	0.04	[0.00, 0.22]	0.04	[0.00, 0.22]	0.01	[0.00, 0.04]
$\varphi _{\beta _{derealization}}$	0.50	[0.03, 1.57]	0.28	[0.01, 1.23]	1.44	[0.61, 2.57]	0.07	[0.00, 0.33]	0.15	[0.09, 0.28]	0.00	[0.00, 0.01]
$\varphi _{\beta _{analgesia}}$	1.22	[0.09, 2.57]	0.68	[0.04, 2.59]	0.73	[0.06, 1.79]	0.10	[0.00, 0.45]	0.10	[0.02, 0.23]	0.02	[0.00, 0.09]
Baseline dissociation as predictor of differences in pre-test levels and changes in state dissociation items												
*Intercepts (predictor of scores before stress induction)*												
$\xi _{\alpha _{depersonalization}}$	0.07	[0.05, 0.10]	0.06	[0.03, 0.09]	0.06	[0.03, 0.09]	0.08	[0.06, 0.11]	0.06	[0.03, 0.09]	0.03	[0.00, 0.06]
$\xi _{\alpha _{somatoform}}$	0.05	[0.02, 0.08]	0.07	[0.04, 0.11]	0.04	[0.02, 0.06]	0.07	[0.05, 0.09]	0.04	[-0.01, 0.09]	0.02	[-0.01, 0.04]
$\xi _{\alpha _{derealization}}$	0.08	[0.06, 0.11]	0.06	[0.03, 0.09]	0.06	[0.04, 0.09]	0.10	[0.08, 0.13]	0.05	[0.02, 0.07]	0.01	[-0.01, 0.02]
$\xi _{\alpha _{analgesia}}$	0.09	[0.06, 0.12]	0.08	[0.04, 0.11]	0.05	[0.03, 0.08]	0.05	[0.03, 0.08]	0.08	[0.05, 0.10]	0.06	[-0.01, 0.13]
*Slopes (predictor of change during stress induction)*												
$\xi _{\beta _{depersonalization}}$	0.02	[-0.01, 0.06]	0.01	[-0.02, 0.05]	0.02	[-0.02, 0.06]	0.00	[-0.02, 0.03]	-0.02	[-0.07, 0.03]	-0.03	[-0.06, 0.00]
$\xi _{\beta _{somatoform}}$	**0.04**	**[0.02, 0.07]**	0.00	[-0.03, 0.03]	0.02	[-0.01, 0.06]	-0.01	[-0.03, 0.01]	0.04	[-0.01, 0.09]	0.00	[-0.03, 0.03]
$\xi _{\beta _{derealization}}$	**0.03**	**[0.01, 0.05]**	0.02	[-0.01, 0.05]	0.03	[-0.01, 0.07]	-0.02	[-0.05, 0.00]	0.03	[-0.01, 0.06]	-0.01	[-0.02, 0.01]
$\xi _{\beta _{analgesia}}$	0.00	[-0.02, 0.03]	-0.01	[-0.04, 0.03]	0.03	[-0.01, 0.06]	0.00	[-0.02, 0.02]	-0.04	[-0.07, 0.00]	-0.04	[-0.07, -0.01]
Amount of variance explained by baseline dissociation in pre-test levels and changes in state dissociation items (${R^2}$)												
*Intercepts (variance explained in scores before stress induction)*												
${}_{\alpha _{depersonalization}}$	0.37	[0.14, 0.65]	0.22	[0.06, 0.45]	0.14	[0.03, 0.32]	0.33	[0.17, 0.49]	0.18	[0.03, 0.40]	0.17	[0.01, 0.52]
${}_{\alpha _{somatoform}}$	0.14	[0.03, 0.32]	0.20	[0.06, 0.38]	0.23	[0.07, 0.51]	0.33	[0.16, 0.54]	0.05	[0.00, 0.22]	0.04	[0.00, 0.26]
${}_{\alpha _{derealization}}$	0.31	[0.15, 0.48]	0.17	[0.04, 0.36]	0.31	[0.11, 0.63]	0.44	[0.27, 0.60]	0.25	[0.06, 0.51]	0.04	[0.00, 0.32]
${}_{\alpha _{analgesia}}$	0.23	[0.10, 0.38]	0.20	[0.05, 0.39]	0.15	[0.04, 0.33]	0.16	[0.05, 0.31]	0.38	[0.14, 0.66]	0.04	[0.00, 0.17]
*Slopes (variance explained in change during stress induction)*												
${}_{\beta _{depersonalization}}$	0.10	[0.00, 0.72]	0.10	[0.00, 0.88]	0.03	[0.00, 0.23]	0.05	[0.00, 0.64]	0.02	[0.00, 0.12]	0.54	[0.00, 0.97]
${}_{\beta _{somatoform}}$	**0.45**	**[0.10, 0.96]**	0.05	[0.00, 0.65]	0.02	[0.00, 0.11]	0.17	[0.00, 0.90]	0.22	[0.00, 0.88]	0.10	[0.00, 0.75]
${}_{\beta _{derealization}}$	**0.26**	**[0.02, 0.90]**	0.14	[0.00, 0.88]	0.06	[0.00, 0.27]	0.36	[0.00, 0.96]	0.05	[0.00, 0.20]	0.11	[0.00, 0.73]
${}_{\beta _{analgesia}}$	0.01	[0.00, 0.30]	0.04	[0.00, 0.66]	0.07	[0.00, 0.61]	0.05	[0.00, 0.73]	0.11	[0.00, 0.50]	0.45	[0.02, 0.95]

*Note.* Results particularly relevant to our hypothesis test are in bold. All parameters are unstandardized and denote posterior medians. The 95% CIs denote Bayesian credibility intervals. ${R^2}$ measures refer to explained variance on the between level. Grandmean centered predictor. $\xi$ = regression coefficients of regressing the respective parameter on baseline dissociation values, ${\alpha }$ = average intercept (fixed effect) of dissociative states items at time point 1 before the stress induction, ${\beta }$ = average slope (fixed effect) of linear change in dissociative states items from time point 1 before the stress induction to time point 2 after the stress induction, $\varphi _{\alpha }$ = random, person-specific intercept, $\varphi _{\beta }$ = random, person-specific slope.

We used M*plus* 8.8 [54] to estimate the models for our hypothesis tests, and for additional analyses. Preliminary analyses and all other reliability estimates were computed using R Version 4.2.1 [55].

## Results

### Baseline measures

Prior to the main analysis, we assessed baseline group differences in dissociation, childhood maltreatment, and severity of depressive symptoms. The groups significantly differed in all three baseline measures. The BPD/PTSD group displayed the highest baseline dissociation and maltreatment scores, followed by the MDD group, and the NCC group displayed the lowest scores. In line with other studies, the BPD/PTSD group displayed higher depression scores than NCCs as well as patients with MDD [21, 56]. For means, standard deviations and test statistics, see Table 1. For post-hoc t-test results, see Table 1. legend.

#### Treatment check: affective response to stress

Detailed results on affective and physiological stress responses, are reported in the previous publications [34–36]. For the present analysis, we investigated group differences in changes in three affective states during the TSST and P-TSST. Repeated measures ANOVA showed a significant main effect of group, suggesting group differences in positive–negative mood in the TSST condition, F(2,188) = 76.48, *p* < 0.001 (BPD/PTSD = MDD > NCC), and the P-TSST condition, F(2,187) = 28.62, *p* < 0.001 (BPD/PTSD = MDD > NCC), differences in awake-tired mood in the TSST condition, F(2,188) = 26.40, *p* < 0.001 (BPD/PTSD = MDD > NCC), and the P-TSST condition, F(2,187) = 11.09, *p* < 0.001 (BPD/PTSD = MDD > NCC), and group differences in calm-agitated mood in the TSST condition, F(2,188) = 59.15, *p* < 0.001 (BPD/PTSD = MDD > NCC), and the P-TSST condition, F(2,187) = 22.32, *p* < 0.001 (BPD/PTSD = MDD > NCC). For means, standard deviations, effect sizes, confidence intervals and full post hoc results see supplement material S5.

As a treatment check, we compared affective states before and after (P-)TSST. After TSST, participants reported more negative affect, feeling more tired, and more agitated, than before. After P-TSST, participants reported more positive affect, feeling more awake, and calmer, than before. These results indicate that the stress manipulation was successful. For detailed results, see supplement material S5.

### Main analyses

#### Change in state dissociation items

**Fig. 2:**
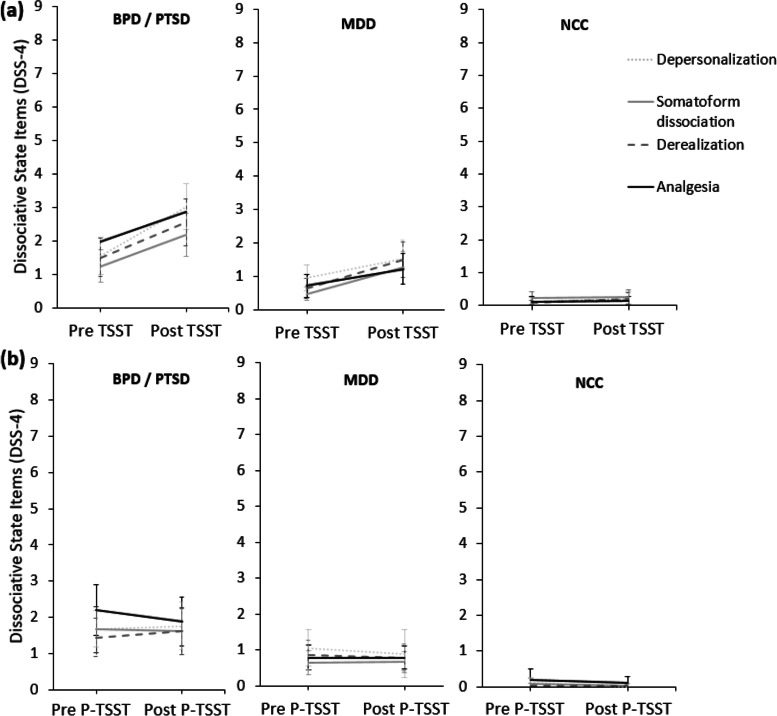
*Mean Scores of State Dissociation Items Across Diagnostic Groups Immediately Before and After the TSST (a) and P-TSST (b).* Error bars display 95% confidence intervals. TSST = Trier Social Stress Test; P-TSST = Placebo Trier Social Stress Test; BPD = borderline personality disorder; PTSD = posttraumatic-stress disorder; MDD = major depressive disorder; NCC = non-clinical controls; DSS-4 = Dissociation Tension Scale acute

As in previous studies, the descriptive results in Fig. 2 show that scores on all four state dissociation items increase from immediately before to immediately after the TSST in patients with PTSD and/or BPD, as well as in patients with MDD but not in NCCs. The point estimates and confidence intervals for the slopes in Table 2 (see second section below “Fixed effects (average effects across participants)” with the title “Slopes (change during stress induction)”) confirm that the increases in dissociative states during the TSST are statistically significant only in the BPD/PTSD and MDD groups but not the NCC group. Dissociative states did not significantly change during the P-TSST in any of the groups (see Fig. 2 and Table 2 second section “slopes (change during stress induction)”). In addition, point estimates and confidence intervals for intercepts in Table 2 (see first section below “Fixed effects (average effects across participants)” with the title “Intercepts (before stress induction)”) show that baseline levels of dissociative states before the TSST or P-TSST are higher in patients with PTSD and/or BPD compared to patients with MDD and NCCs.

#### Dependency of change in state dissociation items on baseline dissociation

We expected larger increases in state dissociation items under stress among patients with PTSD and/or BPD who report higher scores on baseline dissociation. Our results show that differences in baseline dissociation predict changes in the somatoform and derealization state dissociation items but not the depersonalization and analgesia state dissociation items during the TSST in patients with BPD and/or PTSD, but not patients with MDD in the TSST. Specifically, as shown in Table 2 (see second section below “Baseline dissociation as predictor of differences in baseline levels and changes in state dissociation items” with the title “Slopes (predictor of change during stress induction)”), patients with PTSD and/or BPD higher in baseline dissociation report significantly larger changes in the somatoform dissociation item (0.04, 95% CI [0.02, 0.07]) and the derealization item (0.03, 95% CI [0.01, 0.05]) in the TSST condition. There were no significant effects for the depersonalization item (0.02, 95% CI [-0.01, 0.06]) and the analgesia item (0.00, 95% CI [-0.02, 0.03]). Results in Table 2 also show that baseline dissociation scores do not significantly predict changes in any state dissociation item among patients with MDD during the TSST (depersonalization: 0.02, 95% CI [-0.02, 0.06], somatoform dissociation: 0.02, 95% CI [-0.01, 0.06], derealization: 0.03, 95% CI [-0.01, 0.07], analgesia: 0.03, 95% CI [-0.01, 0.06]). In addition, Table 2 (see second section below “Amount of variance explained by baseline dissociation in pre-test levels and changes in state dissociation items (${R}^{2}$)” with the title “Slopes (variance explained in change during stress induction)”), shows that in patients with BPD and/or PTSD differences in baseline dissociation scores explained an estimated 45%, 95% CI [0.10, 0.96], of the total variance in changes in the somatoform dissociation item, and 26%, 95% CI [0.02, 0.90], variance in changes in the derealization item. Finally, results in Table 2 (see second section below “Baseline dissociation as predictor of differences in pre-test levels and changes in state dissociation items” with the title “Intercepts (predictor of scores before stress induction)”) show that patients in the BPD/PTSD and MDD groups higher in baseline dissociation report significantly higher scores in all four state dissociation items before the TSST and before the P-TSST.

### Sensitivity and additional analyses

To test the robustness of our effects and to explore further, we conducted several sensitivity and additional analyses. First, measurement invariance tests were conducted in patients with MDD. Results show that a model assuming configural scale invariance is an inappropriate fit across patients with MDD in the TSST condition, ${\chi }^{2}$(19, *N* = 84) = 84.38, *p* = 0.000, CFI = 0.82, RMSEA = 0.20, 95% CI [0.16, 0.25], SRMR = 0.08, and the P-TSST condition, ${\chi }^{2}$(19, *N* = 84) = 176.13, *p* = 0.000, CFI = 0.68, RMSEA = 0.31, 95% CI [0.27, 0.36], SRMR = 0.10. In addition, we repeated all measurement invariance tests using ordinal indicators, which may be more appropriate [57]. The results using ordinal indicators confirm our main conclusion that the prerequisite of even configural scale invariance is not met (fit in the TSST condition across patients with BPD and/or PTSD, ${\chi }^{2}$(18, *N* = 64) = 66.99, *p* = 0.000, CFI = 0.97, RMSEA = 0.21, 95% CI [0.16, 0.26], SRMR = 0.05, and across patients with MDD, ${\chi }^{2}$(19, *N* = 84) = 55.85, *p* = 0.000, CFI = 0.97, RMSEA = 0.15, 95% CI [0.11, 0.20], SRMR = 0.05). Second, we repeated our main analysis using separate models for patients with BPD and/or PTSD who received the P-TSST before the TSST and those who received the TSST before the P-TSST (cross-over design). Results indicate that baseline dissociation scores significantly predicted changes in somatoform dissociation only among participants who received the P-TSST before the TSST, and changes in derealization only among participants who received the TSST before the P-TSST. Third, we repeated our main analysis using separate models for patients who were only diagnosed with BPD (*n* = 24), patients who were only diagnosed with PTSD (*n* = 20), and patients who were diagnosed with both BPD and PTSD (*n* = 20). Results indicate no significant links between baseline dissociation scores and changes in dissociative states during the TSST when separately investigating the diagnostic groups. The models may lack statistical power to detect effects at this level of analysis. In addition, results show significant increases in all four dissociation items during the TSST in patients with BPD and patients with both BPD and PTSD. However, in patients with PTSD only changes in the derealization item reached significance. Fourth, we repeated our main analysis excluding the two patients with comorbid BPD from the MDD sample. Results indicate only minor changes in parameter estimates and all main conclusions remain the same. Fifth, we repeated our main analysis using the childhood trauma questionnaire scores at baseline as a predictor of changes in dissociative states. Results indicate that patients with BPD and/or PTSD who report higher overall levels of childhood maltreatment (as assessed by the Childhood Trauma Questionnaire) experience significantly higher increases in dissociative states of analgesia during the TSST. Sixth, we repeated our main analysis including changes in affective states in patients with BPD and/or PTSD during the TSST. Results indicate that our main finding (DSS-7 scores significantly relate to increases in DSS-4 somatoform dissociation and depersonalization) remains the same when including changes in calm-agitated mood, positive–negative mood, and awake-tired mood as controls in our statistical models. Sixth, we repeated our main analysis including depressive scores (BDI-II) in patients with BPD and/or PTSD during the TSST. Results indicate that DSS-7 scores significantly relate to increases in DSS-4 depersonalization (but not somatoform dissociation) when including BDI-II scores in our analyses. BDI-II scores did not significantly relate to increases in any DSS-4 item. Seventh, we repeated our main analysis using subscale scores of the DSS-7 reflecting baseline levels of somatoform dissociation (9 items), derealization (3 items), depersonalization (4 items), analgesia (1 item) as predictors. Results indicate the same finding as in our main analysis (significant relations with increases in DSS-4 somatoform dissociation and depersonalization) when using the DSS-7 somatoform dissociation, depersonalization, and analgesia subscale scores. When using the DSS-7 derealization subscale scores, we find no significant relations with any DSS-4 item.

## Discussion

In the present analysis we investigated the relationship between dissociative symptoms in the past week (baseline trait-like dissociation) and state dissociation items in response to stress induced using the Trier Social Stress Test (TSST) in a mixed sample of patients with borderline personality disorder (BPD) and posttraumatic-stress disorder (PTSD) compared to patients with major depressive disorder (MDD) and non-clinical controls (NCC). As expected, we found significant increases in all state dissociation items (i.e., depersonalization, somatoform dissociation, derealization, analgesia) in response to the TSST in patients with BPD and/or PTSD, as well as patients with MDD, but not in NCCs. In addition, increases in two dissociative states (derealization and somatoform dissociation) were significantly related to baseline differences in trait-like dissociation in patients with BPD and/or PTSD, but not patients with MDD and NCCs.

### Psychosocial stress increases state dissociation in patients with BPD/PTSD and MDD

Our results replicate previous studies showing that patients with BPD and/or PTSD who were exposed to a psychosocial stressor report larger increases in stress-related state dissociation than NCCs [25, 26, 31]. We extend this finding by demonstrating that increases in stress-related state dissociation in patients with BPD and/or PTSD are comparable to those observed in patients with MDD (although less pronounced), which corresponds to conceptualizations of dissociative reactions as a transdiagnostic phenomenon [2]. One previous study did not find significant differences in state dissociation change between patients with BPD and NCCs [30]. However, this study measured dissociation 40 min after the stressor and dissociative reactions can be expected to diminish relatively quickly [21]. While results of our additional analyses confirm that patients with BPD, as well as patients with BPD and a comorbid PTSD, show significant increases in all state dissociation items (somatoform dissociation, derealization, depersonalization, analgesia), patients with PTSD only reported significant increases in depersonalization. This suggests that stress-related dissociative reactions can differ across diagnostic categories. For example, it could be that under stress, patients with PTSD are more likely to report psychological dissociation symptoms (e.g., depersonalization), while patients with (comorbid) BPD also report somatoform dissociation symptoms (including analgesia) [58, 59].

### Changes in state dissociation are partially predicted by baseline dissociation

As expected, we found larger increases in dissociative states during the TSST among patients with BPD and/or PTSD who reported higher baseline dissociation scores, which shows that dissociative symptoms in the week before a stressor relate to stress-induced changes in state dissociation. This is in line with current conceptualizations of dissociation in psychopathology [3, 26], and more generally latent state-trait theory [60]. Importantly, changes in state dissociation were not related to baseline dissociation in patients with MDD, indicating that while dissociative reactions can be observed in various mental disorders, the link between severity of past dissociative symptoms and acute dissociative reactions may be specific to BPD and/or PTSD. This finding loosely supports the notion of trauma models that some individuals with a history of maltreatment may develop relatively stable and recurring patterns of dissociative symptoms (trait or trait-like) that are associated with a higher risk of experiencing acute dissociative states in response to (relatively unspecific) psychosocial stressors [16, 20]. One such stressor might be social evaluation, which is known to be one of the most stressful components of the TSST [27, 61]. Our results only support significant relations between baseline dissociation and changes in somatoform state dissociation (e.g., problems hearing) and state derealization in patients with BPD and/or PTSD, but not for state depersonalization and state analgesia. It is possible that differences in the increase in certain facets of dissociation are better predicted by levels of dissociative symptoms in the past week than others. In addition, when including depressive symptom severity into our analysis, the relation between baseline dissociation and changes in somatoform state dissociation no longer reached statistical significance, indicating that this relation could be explained by an overlap between baseline dissociation and general symptom severity.

### Assessment of changes in state dissociation

Findings from this study inform the assessment in changes in state dissociation, especially during a stress induction in patients with PTSD and/or BPD. Generally, investigating change in a psychological construct requires that the construct under investigation is assessed in a way that ensures that a construct has the same (psychometric) structure or meaning on different measurement occasions (i.e., measurement invariance over time) [47, 62]. Results from this study illustrate the consequences of measurement invariance. If the four items of state dissociation are combined into a scale to compare state dissociation change from pre-test to post-test, mean differences on the scale may mislead for example because the stress task impacted the depersonalization item, especially in patients with PTSD, more strongly than the somatoform, derealization, and analgesia items of the state dissociation construct [6]. In addition, measures of baseline dissociation were significantly related to only two of the four items. This also points to the importance of conceptualizing state dissociation as a multifaceted construct – and separately investigating different facets [63]. Existing state dissociation scales should be developed further to fulfill the prerequisite of measurement invariance over time in mixed samples including patients with BPD and/or PTSD, especially when being exposed to an experimental or real-world stressor, because there is growing interest in the investigation of the relationship between stress and state dissociation [64]. A first step towards this goal could be to differentiate between various facets of state dissociation (e.g., states of derealization or depersonalization), which to us makes sense given the multifaceted structure of the dissociation construct [3]. Researchers should start by formulating additional homogenous items to assess the four facets of state dissociation identified as particularly relevant in clinical settings [6, 65]. Afterwards, psychometric properties of the updated scale should be investigated, including formal tests of measurement invariance over time [47].

### Limitations and future research

Several limitations and perspectives for future research should be mentioned. First, we reanalyzed existing data using exploratory hypotheses. Thus, we cannot assume that error rates in our inferences are controlled for as one would expect in confirmatory research and results should be interpreted cautiously [66, 67]. We encourage replications using a priori, pre-registered hypotheses. Second, our measure of baseline dissociation, the Dissociation Tension Scale (DSS-7) [8], assesses dissociative symptoms within the last seven days. Scores may be particularly influenced by recent life events or current general symptom load. Future studies should include a measure of trait dissociation that assesses dissociative experiences in general, for example the Dissociative Experience Scale (DES) [7], which asks participants how often they have experienced various dissociative states throughout their lives. In addition, while the DSS-7 includes different numbers of items assessing the four dissociative states included in the DSS-4, it also includes other items (e.g., “I remembered an event so vividly as if I were reliving it”), which complicates distinguishing effects from psychopathological phenomena other than dissociation such as flashbacks. We encourage future researchers to conceptually distinguish different facets of dissociation, differentiate them from other psychopathological phenomena, and use the same number of items to assess each facet of dissociation [68]. Third, mean DSS-4 scores after stress show low to moderate state dissociation, which is comparable to previous findings of studies using the TSST [31] or other methods of stress induction [69, 70]. These results either suggest that current procedures only induce mild dissociation or that some participants show stronger dissociative reactions than others. Careful inspection of raw data suggest the latter. We recommend that future researchers screen patients who report at least moderate levels of baseline dissociation, which should facilitate investigating substantial dissociative reactions using established procedures. Fourth, our samples consist exclusively of women and findings may not generalize to men. Current evidence suggests that men and women process traumatic events differently. For example, women are more likely to report peri-traumatic dissociation [71, 72] and are more often and more severely affected by PTSD [73, 74]. These findings suggest that women might react to acute stressors with more state dissociation than men. Previous analyses however, found no differences in trait dissociation between males and females [75]. Future research could compare the relation between baseline levels of dissociation and stress-related dissociative reactions across gender categories. Finally, patients with dissociative disorders should be included in future studies. We recommend the use of specific structured clinical interviews to determine the presence or absence of dissociative disorders (e.g., Dissociative Disorders Interview Schedule; [76]).

### Conclusion and clinical implications

In sum, patients with BPD and/or PTSD, as well as patients with MDD, react to acute stress with increased dissociative states (depersonalization, derealization, somatoform dissociation, and analgesia), while non-clinical control participants do not. In patients with BPD and/or PTSD, levels of baseline dissociation in the week prior to the stressor modulate increases in two dissociative states (somatoform dissociation and derealization). Clinicians could use measures of baseline dissociation to facilitate the prediction of stress-induced acute dissociation in patients with BPD and/or PTSD. This could help to administer interventions aiming to reduce dissociative symptoms through modulating states of tension and distress, as well as regulating distressing emotions, as used in evidence-based treatment programs for BPD and PTSD [77, 78].

## Supplementary Information

**Additional file1: S1 **Current Comorbid Diagnoses and Psychotropic Medication in Patients with BPD and/or PTSD (n = 65), and Patients with MDD (n = 84). **S2. ***Type and Frequency of Traumatic Experiences in Patients with BPD and/or PTSD (n *= 65*). ***S3 ***Participant Flow Study 1 –Patients with BPD and/or PTSD and Non-Clinical Controls. ***S4. **Dissociation Tension Scale acute (DSS-4) items. Original German items and English translation.

**Additional file2.**

## Data Availability

The data reported in this manuscript were collected in two separate and previously published studies. Anonymized data sets will be made available to researchers upon request. Statistical code and (additional) results for this manuscript are available at https://osf.io/qnmkf.
