# Supplementary material for: Association between baseline dissociation levels and stress-induced state dissociation in patients with posttraumatic-stress disorder, borderline personality disorder, and major depressive disorder
Source: Borderline Personal Disord Emot Dysregul. 2023 Mar 30;10:11. doi: 10.1186/s40479-023-00215-2 (PMC10064785; doi:10.1186/s40479-023-00215-2)
Supplement: Supplementary file 1 — Additional file1: S1 Current Comorbid Diagnoses and Psychotropic Medication in Patients with BPD and/or PTSD (n = 65), and Patients with MDD (n = 84). S2. Type and Frequency of Traumatic Experiences in Patients with BPD and/or PTSD (n = 65). S3 Participant Flow Study 1 –Patients with BPD and/or PTSD and Non-Clinical Controls. S4. Dissociation Tension Scale acute (DSS-4) items. Original German items and English translation. [file 40479_2023_215_MOESM1_ESM.pdf]

Table 2

Results of Structural Equation Models of Baseline Levels and Changes in Dissociative States Items in Stress and Non-Stress Conditions in Patient and Non-Clinical Samples Predicted by Baseline Dissociation

	Borderline Personality and/or Posttraumatic Stress Disorder				Major Depressive Disorder				Non-Clinical Controls			
	Stress Condition (TSST)		Non-Stress Condition (P-TSST)		Stress Condition (TSST)		Non-Stress Condition (P-TSST)		Stress Condition (TSST)		Non-Stress Condition (P-TSST)	
	n = 64		n = 62		n = 84		n = 84		n = 43		n = 44	
	Estimate	95% CI	Estimate	95% CI	Estimate	95% CI	Estimate	95% CI	Estimate	95% CI	Estimate	95% CI
Fixed effects (average effects across participants)												
Intercepts (before stress induction)												
$\gamma_{\alpha_{depersonalization}}$	1.54	[1.06, 2.08]	1.67	[1.13, 2.22]	0.93	[0.51, 1.34]	1.04	[0.72, 1.35]	0.12	[-0.02, 0.25]	0.11	[0.01, 0.22]
$\gamma_{\alpha_{somatoform}}$	1.26	[1.06, 2.08]	1.67	[1.08, 2.24]	0.49	[0.25, 0.71]	0.69	[0.40, 0.97]	0.23	[0.02, 0.45]	0.09	[-0.02, 0.20]
$\gamma_{\alpha_{derealization}}$	1.51	[1.02, 1.99]	1.43	[0.90, 1.97]	0.66	[0.33, 0.99]	0.96	[0.65, 1.27]	0.07	[-0.02, 0.17]	0.05	[-0.02, 0.11]
$\gamma_{\alpha_{analgesia}}$	1.98	[1.42, 2.56]	2.19	[1.56, 2.86]	0.75	[0.40, 1.10]	0.85	[0.53, 1.16]	0.12	[-0.01, 0.24]	0.20	[-0.07, 0.47]
Slopes (change during stress induction)												
$\gamma_{\beta_{depersonalization}}$	1.46	[0.87, 2.07]	0.12	[-0.50, 0.67]	0.69	[0.19, 1.18]	-0.09	[-0.40, 0.21]	0.09	[-0.12, 0.31]	-0.11	[-0.23, 0.01]
$\gamma_{\beta_{somatoform}}$	0.92	[0.43, 1.41]	-0.08	[-0.60, 0.47]	0.86	[0.43, 1.27]	-0.03	[-0.32, 0.26]	0.03	[-0.17, 0.22]	-0.05	[-0.17, 0.08]
$\gamma_{\beta_{derealization}}$	1.04	[0.59, 1.48]	0.17	[-0.37, 0.67]	0.94	[0.47, 1.42]	-0.15	[-0.43, 0.15]	0.14	[-0.01, 0.28]	-0.02	[-0.10, 0.06]
$\gamma_{\beta_{analgesia}}$	0.87	[0.45, 1.30]	-0.26	[-0.90, 0.36]	0.61	[0.22, 1.01]	-0.06	[-0.34, 0.24]	0.02	[-0.13, 0.18]	-0.09	[-0.24, 0.05]
Random-effects variances (differences across participants)												
Intercepts (before stress induction)												
$\varphi_{\alpha_{depersonalization}}$	1.54	[0.56, 3.11]	2.07	[1.00, 3.78]	1.80	[0.90, 3.07]	1.18	[0.70, 1.90]	0.15	[0.06, 0.28]	0.04	[0.01, 0.10]
$\varphi_{\alpha_{somatoform}}$	2.21	[1.19, 3.81]	3.38	[1.92, 5.68]	0.47	[0.18, 0.89]	0.76	[0.39, 1.28]	0.29	[0.14, 0.57]	0.05	[0.02, 0.12]
$\varphi_{\alpha_{derealization}}$	2.54	[1.53, 4.24]	2.57	[1.43, 4.45]	0.73	[0.24, 1.48]	1.15	[0.69, 1.80]	0.06	[0.02, 0.12]	0.01	[0.00, 0.03]
$\varphi_{\alpha_{analgesia}}$	4.52	[2.94, 7.18]	3.90	[2.14, 6.69]	1.42	[0.76, 2.38]	1.28	[0.77, 1.98]	0.08	[0.03, 0.19]	0.72	[0.43, 1.28]
Slopes (change during stress induction)												
$\varphi_{\beta_{depersonalization}}$	0.85	[0.05, 2.64]	0.29	[0.01, 1.30]	1.58	[0.45, 3.04]	0.12	[0.01, 0.50]	0.38	[0.20, 0.67]	0.01	[0.00, 0.04]
$\varphi_{\beta_{somatoform}}$	0.47	[0.03, 1.67]	0.33	[0.02, 1.55]	2.27	[1.45, 3.42]	0.04	[0.00, 0.22]	0.04	[0.00, 0.22]	0.01	[0.00, 0.04]
$\varphi_{\beta_{derealization}}$	0.50	[0.03, 1.57]	0.28	[0.01, 1.23]	1.44	[0.61, 2.57]	0.07	[0.00, 0.33]	0.15	[0.09, 0.28]	0.00	[0.00, 0.01]
$\varphi_{\beta_{analgesia}}$	1.22	[0.09, 2.57]	0.68	[0.04, 2.59]	0.73	[0.06, 1.79]	0.10	[0.00, 0.45]	0.10	[0.02, 0.23]	0.02	[0.00, 0.09]
Baseline dissociation as predictor of differences in pre-test levels and changes in state dissociation items												
Intercepts (predictor of scores before stress induction)												
$\xi_{\alpha_{depersonalization}}$	0.07	[0.05, 0.10]	0.06	[0.03, 0.09]	0.06	[0.03, 0.09]	0.08	[0.06, 0.11]	0.06	[0.03, 0.09]	0.03	[0.00, 0.06]
$\xi_{\alpha_{somatoform}}$	0.05	[0.02, 0.08]	0.07	[0.04, 0.11]	0.04	[0.02, 0.06]	0.07	[0.05, 0.09]	0.04	[-0.01, 0.09]	0.02	[-0.01, 0.04]
$\xi_{\alpha_{derealization}}$	0.08	[0.06, 0.11]	0.06	[0.03, 0.09]	0.06	[0.04, 0.09]	0.10	[0.08, 0.13]	0.05	[0.02, 0.07]	0.01	[-0.01, 0.02]
$\xi_{\alpha_{analgesia}}$	0.09	[0.06, 0.12]	0.08	[0.04, 0.11]	0.05	[0.03, 0.08]	0.05	[0.03, 0.08]	0.08	[0.05, 0.10]	0.06	[-0.01, 0.13]
Slopes (predictor of change during stress induction)												
$\xi_{\beta_{depersonalization}}$	0.02	[-0.01, 0.06]	0.01	[-0.02, 0.05]	0.02	[-0.02, 0.06]	0.00	[-0.02, 0.03]	-0.02	[-0.07, 0.03]	-0.03	[-0.06, 0.00]
$\xi_{\beta_{somatoform}}$	**0.04**	**[0.02, 0.07]**	0.00	[-0.03, 0.03]	0.02	[-0.01, 0.06]	-0.01	[-0.03, 0.01]	0.04	[-0.01, 0.09]	0.00	[-0.03, 0.03]
$\xi_{\beta_{derealization}}$	**0.03**	**[0.01, 0.05]**	0.02	[-0.01, 0.05]	0.03	[-0.01, 0.07]	-0.02	[-0.05, 0.00]	0.03	[-0.01, 0.06]	-0.01	[-0.02, 0.01]
$\xi_{\beta_{analgesia}}$	0.00	[-0.02, 0.03]	-0.01	[-0.04, 0.03]	0.03	[-0.01, 0.06]	0.00	[-0.02, 0.02]	-0.04	[-0.07, 0.00]	-0.04	[-0.07, -0.01]
Amount of variance explained by baseline dissociation in pre-test levels and changes in state dissociation items (R^2)												
Intercepts (variance explained in scores before stress induction)												
$\alpha_{depersonalization}$	0.37	[0.14, 0.65]	0.22	[0.06, 0.45]	0.14	[0.03, 0.32]	0.33	[0.17, 0.49]	0.18	[0.03, 0.40]	0.17	[0.01, 0.52]
$\alpha_{somatoform}$	0.14	[0.03, 0.32]	0.20	[0.06, 0.38]	0.23	[0.07, 0.51]	0.33	[0.16, 0.54]	0.05	[0.00, 0.22]	0.04	[0.00, 0.26]
$\alpha_{derealization}$	0.31	[0.15, 0.48]	0.17	[0.04, 0.36]	0.31	[0.11, 0.63]	0.44	[0.27, 0.60]	0.25	[0.06, 0.51]	0.04	[0.00, 0.32]
$\alpha_{analgesia}$	0.23	[0.10, 0.38]	0.20	[0.05, 0.39]	0.15	[0.04, 0.33]	0.16	[0.05, 0.31]	0.38	[0.14, 0.66]	0.04	[0.00, 0.17]
Slopes (variance explained in change during stress induction)												
$\beta_{depersonalization}$	0.10	[0.00, 0.72]	0.10	[0.00, 0.88]	0.03	[0.00, 0.23]	0.05	[0.00, 0.64]	0.02	[0.00, 0.12]	0.54	[0.00, 0.97]
$\beta_{somatoform}$	**0.45**	**[0.10, 0.96]**	0.05	[0.00, 0.65]	0.02	[0.00, 0.11]	0.17	[0.00, 0.90]	0.22	[0.00, 0.88]	0.10	[0.00, 0.75]
$\beta_{derealization}$	**0.26**	**[0.02, 0.90]**	0.14	[0.00, 0.88]	0.06	[0.00, 0.27]	0.36	[0.00, 0.96]	0.05	[0.00, 0.20]	0.11	[0.00, 0.73]
$\beta_{analgesia}$	0.01	[0.00, 0.30]	0.04	[0.00, 0.66]	0.07	[0.00, 0.61]	0.05	[0.00, 0.73]	0.11	[0.00, 0.50]	0.45	[0.02, 0.95]

Note. Results particularly relevant to our hypothesis test are in bold. All parameters are unstandardized and denote posterior medians. The 95% CIs denote Bayesian credibility intervals. R^2 measures refer to explained variance on the between level. Grandmean centered predictor. ξ = regression coefficients of regressing the respective parameter on baseline dissociation values, α = average intercept (fixed effect) of dissociative states items at time point 1 before the stress induction, β = average slope (fixed effect) of linear change in dissociative states items from time point 1 before the stress induction to time point 2 after the stress induction, φ_α = random, person-specific intercept, φ_β = random, person-specific slope.
