# Supplementary material for: Association between baseline dissociation levels and stress-induced state dissociation in patients with posttraumatic-stress disorder, borderline personality disorder, and major depressive disorder
Source: Borderline Personal Disord Emot Dysregul. 2023 Mar 30;10:11. doi: 10.1186/s40479-023-00215-2 (PMC10064785; doi:10.1186/s40479-023-00215-2)
Supplement: Supplementary file 2 — Additional file2. [file 40479_2023_215_MOESM2_ESM.docx]

Online Supplement

**S1**

*Current Comorbid Diagnoses and Psychotropic Medication in Patients with BPD and/or PTSD (n = 65), and Patients with MDD (n = 84)*

|  | BPD/PTSD | | MDD | |
| --- | --- | --- | --- | --- |
|  | *n* | % | *n* | % |
| **Current comorbid diagnoses according to DSM-IV** |  |  |  |  |
| Obsessive compulsive disorder | 8 | 12.31 | 0 | 0 |
| Social phobia | 8 | 12.31 | 10 | 11.9 |
| Panic disorder | 7 | 10.77 | 6 | 7.14 |
| Agoraphobia with panic disorder | 6 | 9.23 | 3 | 3.57 |
| Generalized anxiety disorder | 3 | 4.62 | 0 | 0 |
| Bulimia nervosa | 3 | 4.62 | 0 | 0 |
| Agoraphobia | 2 | 3.08 | 0 | 0 |
| Alcohol abuse | 1 | 1.54 | 0 | 0 |
| Drug abuse | 1 | 1.54 | 0 | 0 |
| Attention deficit-hyperactivity disorder | 1 | 1.54 | 0 | 0 |
| Specific phobia | 1 | 1.54 | 0 | 0 |
| Somatoform disorder | 1 | 1.54 | 5 | 5.95 |
| Dissociative disorder | 1 | 1.54 | 0 | 0 |
| Dysthymia | 0 | 0 | 9 | 10.71 |
| **Psychotropic medication** |  |  |  |  |
| Selective serotonin reuptake inhibitors (SSRI) | 18 | 27.69 | 15 | 17.86 |
| Anti-psychotics | 13 | 20.00 | 1 | 1.19 |
| Serotonin and noradrenaline reuptake inhibitors (SNRI) | 8 | 12.31 | 10 | 11.9 |
| Anticonvulsants | 8 | 12.31 | 1 | 1.19 |
| Tricyclic antidepressants | 5 | 7.69 | 4 | 4.76 |
| Dopamine and noradrenergic reuptake inhibitors (NDRI) | 4 | 6.15 | 1 | 1.19 |
| Noradrenergic and specific serotonergic antidepressants (NaSSa) | 3 | 4.62 | 9 | 10.71 |

*Note.* BPD = borderline personality disorder; PTSD = posttraumatic-stress disorder; MDD = major depressive disorder; DSM-IV = Diagnostic and Statistical Manual of Mental Disorders 4^th^ edition

**S2**

*Type and Frequency of Traumatic Experiences in Patients with BPD and/or PTSD (n* = 65*)*

| **Type of trauma** |  |  | **Frequency** | | |
| --- | --- | --- | --- | --- | --- |
|  | *n* | % | one time | multiple times | no information |
| Heavy accident | 29 | 44.62 | 17 | 11 | 1 |
| Natural disaster | 5 | 7.69 | 4 | 1 | 0 |
| Violent attack family/ acquaintances | 34 | 53.31 | 4 | 24 | 6 |
| Violent attack stranger | 25 | 38.46 | 9 | 15 | 1 |
| Sexual abuse family/ acquaintances | 30 | 46.15 | 2 | 19 | 9 |
| Sexual abuse stranger | 31 | 47.69 | 20 | 7 | 4 |
| Combat mission | 3 | 4.62 | 1 | 2 | 0 |
| Sexual contact underage | 43 | 66.15 | 9 | 21 | 13 |
| Physical punishment childhood | 30 | 46.15 | 1 | 19 | 10 |
| Captivity | 5 | 7.69 | 2 | 0 | 3 |
| Torture | 4 | 6.15 | 2 | 0 | 2 |
| Life-threatening illness | 11 | 16.92 | 5 | 2 | 4 |
| Other traumatic incident | 38 | 58.46 | 7 | 14 | 15 |

*Note.* Assessed with the German version of the revised Posttraumatic Diagnostic Scale (PDS-R; Foa et al., 1997; Ehlers, et al. 1996); BPD = borderline personality disorder; PTSD = posttraumatic-stress disorder

**S3**

*Participant Flow Study 1 –Patients with BPD and/or PTSD and Non-Clinical Controls*


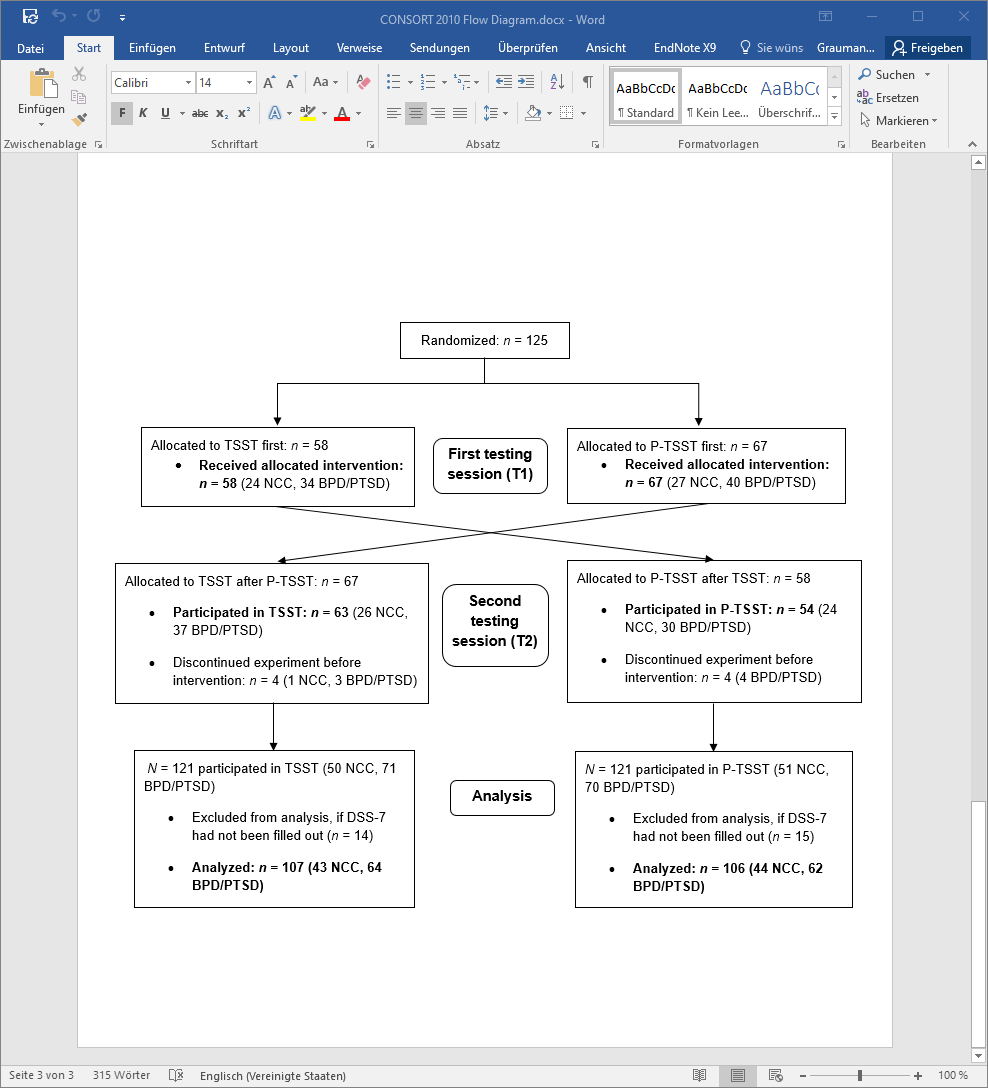


*Note*. TSST = Trier Social Stress Test; P-TSST = Placebo Trier Social Stress Test; NCC = non-clinical controls; BPD = borderline personality disorder; PTSD = posttraumatic-stress disorder; DSS-7 = Dissociation Tension Scale

The original study sample of study 1 (*n* = 125) consisted of 74 female patients (28 BPD, 23 BPD & PTSD, 23 PTSD) and 51 female NCC. Participants were randomly allocated to either the TSST (*n* = 58) in the first session and then to P-TSST in the second session or the P-TSST (*n* = 67) in the first session and TSST in the second session. All allocated participants (*n* = 58) completed the TSST on the first session. Four patients dropped out before the second session and did not participate in the P-TSST. All allocated participants (*n* = 67) participated in the P-TSST on the first session. Three patients and one NCC dropped out before participating in the TSST. Three patients and one NCC dropped out before completion of the TSST. For the present analysis, we excluded 16 participants (nine patients and seven NCC) because they did not answer the Dissociation Tension Scale (DSS-7; Stiglmayr et al., 2010).

*Participant Flow Study 2 –Patients with MDD*

*
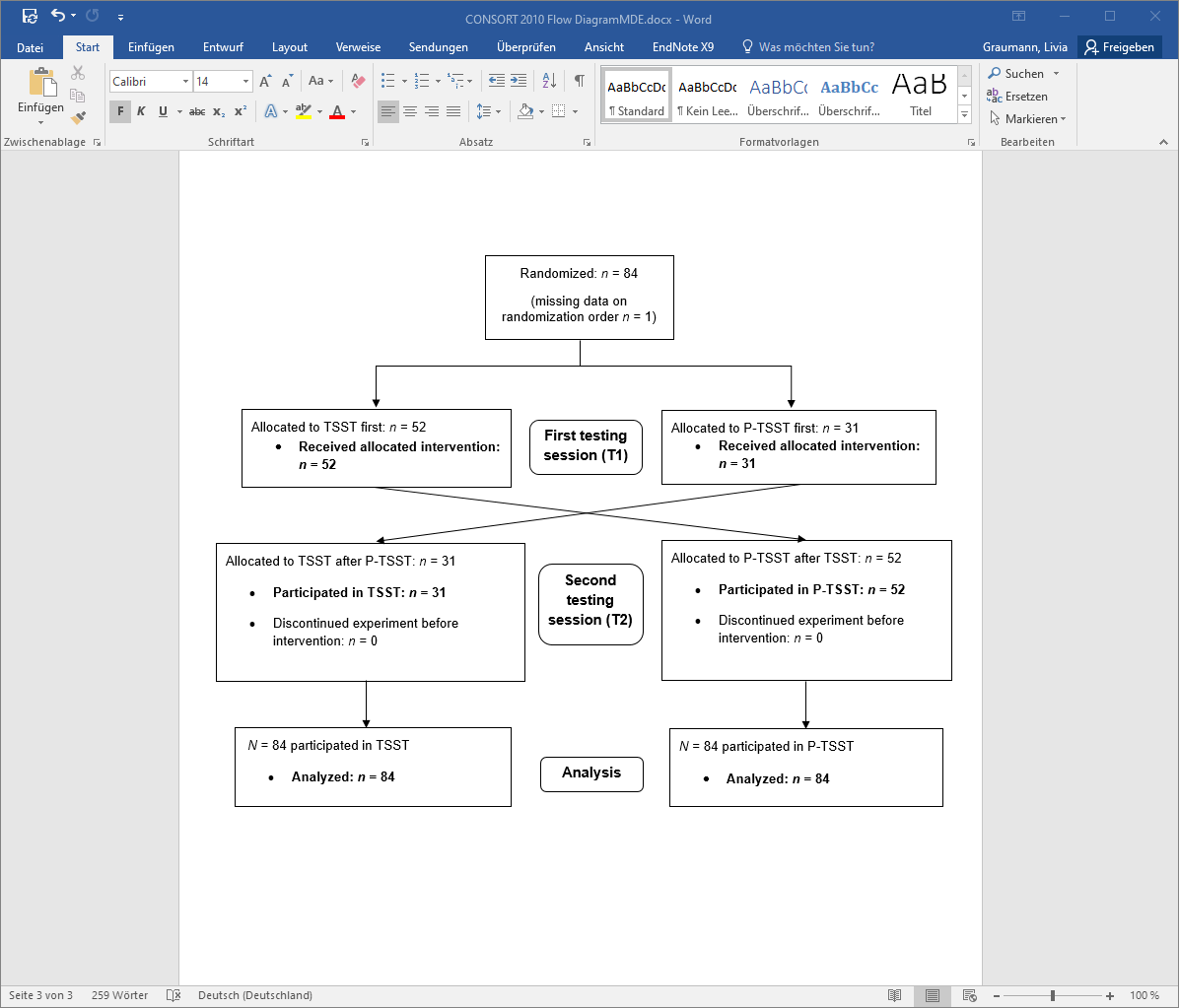
*

*Note*. MDD = major depressive disorder; *n* = sample size; TSST = Trier Social Stress Test; P-TSST = Placebo Trier Social Stress Test

The original study sample comprised 84 female patients with MDD. Participants were randomly allocated to either the TSST (*n* = 52) in the first session and then to P-TSST in the second session or the P-TSST (*n* = 31) in the first session and TSST in the second session. All allocated participants completed the TSST or P-TSST in the first session respectively. In the second session, 31 participants were allocated to and completed the TSST and 52 participants were allocated to and completed the P-TSST. There were no dropouts.

**S4**

Dissociation Tension Scale acute (DSS-4) items. Original German items and English translation.

At this moment…

1. “I have the impression that my body does not belong to me” (German wording: “habe ich die Empfindung; als ob mein Körper nicht zu mir gehört”) (depersonalization)
2. “I have problems hearing; e.g. I hear sounds from nearby as if they come from far away” (German wording: “habe ich Probleme richtig zu hören; z.B. höre ich die Geräusche um mich herum; als ob sie von weit weg kommen”) (somatoform dissociation)
3. “I have the impression other people or things around me are unreal” (German wording: „habe ich die Empfindung; andere Menschen oder andere Dinge oder die Welt um mich herum seien nicht wirklich“) (derealization)
4. „I have the impression that my body or parts of it are insensitive to pain” (German wording: „habe ich die Empfindung; daß mein Körper oder einzelne Teile davon unempfindlich gegenüber körperlichen Schmerzen sind“) (analgesia)

**S5**

*Mood States Before and After (P-)TSST in Women with BPD/PTSD, MDD and NCC*

| **Variable**  *M (SD)* | **BPD/PTSD** | **MDD** | **NCC** | **Statistics** |
| --- | --- | --- | --- | --- |
| **Positive vs. Negative** |  |  |  | **Time:** *F*(1,188) = 83.49, *p* < .001, *η ^2^* = 0.31, 90% CI [0.22, 0.39] |
| Pre TSST | 12.19 (3.97) | 10.77 (1.25) | 17.23 (2.27) | **Time** × **group:** *F*(2,188) = 6.27, *p* = .002, *η^2^* = 0.06, 90% CI [0.01, 0.12] |
| Post TSST | 8.45 (4.09) | 9.31 (3.94) | 14.33 (2.97) | **Group:** *F*(2,188) = 76.48, *p* < .001, *η^2^* = 0.45, 90% CI [0.36, 0.52] |
| Pre P-TSST | 12.26 (3.93) | 12.44 (3.23) | 16.45 (2.81) | **Time:** *F*(1,187) = 17.85, *p* < .001, *η^2^* = 0.09, 90% CI [0.03, 0.16] |
| Post P-TSST | 13.58 (4.04) | 13.30 (3.24) | 17.16 (2.34) | **Time** × **group:** *F*(2,187) = 0.65, *p* = .525, *η^2^* = 0.01, 90% CI [0.00, 0.03] |
|  |  |  |  | **Group:** *F*(2,187) = 28.62, *p* < .001, *η^2^* = 0.23, 90% CI [0.15, 0.31] |
| **Awake vs. Tired** |  |  |  | **Time:** *F*(1,188) = 5.67, *p* = .018, *η^2^* = 0.03, 90% CI [0.00, 0.07] |
| Pre TSST | 9.77 (4.02) | 11.07 (3.86) | 14.02 (3.31) | **Time** × **group**: *F*(2,188) = 0.30, *p* = .741, *η^2^* = 0.00, 90% CI [0.00, 0.02] |
| Post TSST | 8.89 (3.47) | 10.44 (3.43) | 13.67 (3.34) | **Group**: *F*(2,188) = 26.40, *p* < .001, *η ^2^*= 0.22, 90% CI [0.133, 0.30] |
| Pre P-TSST | 10.32 (3.75) | 10.89 (3.81) | 13.34 (3.09) | **Time:** *F*(1,187) = 7.47, *p* = .007, *η ^2^*= 0.04, 90% CI [0.01, 0.09] |
| Post P-TSST | 10.94 (3.87) | 11.56 (3.58) | 13.80 (2.83) | **Time** × **group:** *F*(2,187) = 0.08, *p* = .920, *η ^2^*= 0.00, 90% CI [0.00, 0.01] |
|  |  |  |  | **Group**: *F*(2,187) = 11.09, *p* < .001, *η ^2^*= 0.11, 90% CI [0.04, 0.17] |
| **Calm vs. Nervous** |  |  |  | **Time**: *F*(1,188) = 87.45, *p* < .001, *η ^2^*= 0.32, 90% CI [0.23, 0.40] |
| Pre TSST | 11.22 (3.39) | 10.63 (1.92) | 15.35 (2.41) | **Time** × **group**: *F*(2,188) = 1.51, *p* = .223, *η ^2^*= 0.02, 90% CI [0.00, 0.05] |
| Post TSST | 8.27 (3.52) | 8.68 (3.04) | 12.67 (2.97) | **Group**: *F*(2,188) = 59.15, *p* < .001, *η^2^* = 0.39, 90% CI [0.29, 0.46] |
| Pre P-TSST | 10.55 (3.47) | 11.80 (3.15) | 14.91 (3.38) | **Time**: *F*(1,187) = 9.31, *p* = .003, *η^2^* = 0.05, 90% CI [0.01, 0.11] |
| Post P-TSST | 11.98 (4.24) | 12.14 (3.25) | 15.31 (3.16) | **Time** × **group:** *F*(2,187) = 2.36, *p* = .098, *η ^2^*= 0.03, 90% CI [0.00, 0.07] |
|  |  |  |  | **Group**: *F*(2,187) = 22.32, *p* < .001, *η^2^* = 0.19, 90% CI [0.11, 0.27] |

Note. PTSD = posttraumatic-stress disorder; BPD = borderline personality disorder; MDD = major depressive disorder; NCC = non-clinical controls; TSST = Trier Social Stress Test; P-TSST = Placebo Trier Social Stress Test*; η ^2^*= effect size; CI = confidence interval

***Affective Responses to TSST and P-TSST***

After TSST, participants reported more negative affect, *F*(1,188) = 83.49 , *p* < .001, *η ^2^* = 0.31, 90% CI [0.22, 0.39], feeling more tired, *F*(1,188) = 5.67, *p* = .018, *η^2^* = 0.03, 90% CI [0.00, 0.07], and more agitated, *F*(1,188) = 87.45, *p* < .001, *η ^2^*= 0.32, 90% CI [0.23, 0.40], than before (time effect). After P-TSST, participants reported more positive affect, *F*(1,187) = 17.85 , *p* < .001, *η^2^* = 0.09, 90% CI [0.03, 0.16], feeling more awake, *F*(1,187) = 7.47, *p* = .007, *η ^2^*= 0.04, 90% CI [0.01, 0.09], and calmer, *F*(1,187) = 9.31, *p* = .003, *η^2^* = 0.05, 90% CI [0.01, 0.011], than before (time effect).

***Post Hoc Tests***

**Positive-Negative Mood.** There was a time × group interaction in the TSST condition, *F*(2,188) = 6.27, *p* = .002, *η^2^* = 0.06, 90% CI [0.01, 0.12]. Post hoc t-tests with alpha corrected for multiple testing revealed that before TSST, mood was significantly more negative in the MDD group than the BPD/PTSD group, *t*(146) = -3.07, *p* = .003, *d* = -0.51, 95% CI [-0.84, -0.18], while there were no significant differences between both patient groups after TSST, *t*(146) = 0.77, *p* = .45, *d* = 0.21, 95% CI [-0.11, 0.54]. Before TSST, the NCC group reported more positive affect than the BPD/PTSD group, *t*(105) = -7.54, *p* < .001, *d* = -1.49, 95% CI [-1.92, -1.05], and the MDD group, *t*(125) = 20.69, *p* < .001, *d* = 3.88, 95% CI [3.27, 4.48]. After TSST, the NCC group reported more positive affect than the BPD/PTSD group, *t*(105) = -8.10, *p* < .001, *d* = -1.60, 95% CI [-2.04, -1.15], and the MDD group, *t*(125) = 7.35, *p* < .001, *d* = 1.38, 95% CI [0.97, 1.78].

Post hoc t-tests revealed that both patient groups reported worse mood than NCCs before and after P-TSST, all *p*s < .001 and there were no differences between both patient groups in reported mood before, *p* = .759, or after P-TSST, *p* = .640.

**Awake vs. Tired Mood.** Post hoc t-tests showed that before the TSST, NCCs reported feeling less tired than patients with BPD/PTSD, *t*(105) = -5.76, *p* < .001, *d* = -1.14, 95% CI [-1.55, -7.2], and patients with MDD, *t*(125) = 4.28, *p* < .001, *d* = 0.80, 95% CI [0.42, 1.18]. There were no differences between patient groups in feelings of alertness before TSST, *t*(146) = -2.00, *p* = .047, *d* = -0.33, 95% CI [-0.66, -0.00]. After TSST, NCCs reported feeling less tired than patients with BPD/PTSD, *t*(105) = -7.09, *p* < .001, *d* = -1.40, 95% CI [-1.83, -0.97], and patients with MDD, *t*(125) = 5.07, *p* < .001, *d* = 0.95, 95% CI [0.56, 1.34] Patient groups did not significantly differ in alertness after TSST, *t*(146) = -2.71, *p* = .008, *d* = -0.45, 95% CI [-0.78, -0.12]

Before and after P-TSST, both patient groups reported feeling more tired than NCCs, all *p*s < .001 and there were no differences between patient groups before P-TSST, *p* = .369, or after P-TSST, *p* = .317.

**Calm vs. Agitated Mood.** Post hoc t-tests showed that before the TSST, NCCs reported feeling calmer than patients with BPD/PTSD, *t*(105) = -6.90, *p* < .001, *d* = -1.36, 95% CI [-1.79, -0.93], and patients with MDD, *t*(125) = 11.98, *p* < .001, *d* = 2.25, 95% CI [1.78, 2.70]. There were no differences between patient groups in feelings of agitation before TSST, *t*(146) = 1.33, *p* = .185, *d* = 0.22, 95% CI [-0.11, 0.55]. After TSST, NCCs reported feeling calmer than patients with BPD/PTSD, *t*(105) = -6.78, *p* < .001, *d* = -1.33, 95% CI [-1.77, -0.90], and patients with MDD, *t*(125) = 7.07, *p* < .001, *d* = 1.33, 95% CI [0.92, 1.73]. There were no differences between patient groups in feelings of agitation after TSST, *t*(146) = --0.77, *p* = .446, *d* = -0.13, 95% CI [-0.45, 0.20].

Before and after P-TSST, both patient groups reported feeling more nervous than NCCs, all *p*s < .001, and there were no differences between patient groups before P-TSST, *p* = .025, or after, *p* = .798.
